# Supplementary material for: Immune Checkpoint Inhibitor-Related Cytopenias: About 68 Cases from the French Pharmacovigilance Database
Source: Cancers (Basel). 2022 Oct 14;14(20):5030. doi: 10.3390/cancers14205030 (PMC9599380; doi:10.3390/cancers14205030)
Supplement: Supplementary file 1 [file cancers-14-05030-s001.zip › cancers-1925419-SI.pdf]

cancers MDPI

Supplementary Materials

Immune Checkpoint Inhibitor-Related Cytopenias: About 68 Cases from the French Pharmacovigilance Database

Mickaël Martin [1,2,3,*], Hoan-My Nguyen [1], Clément Beuvon [1,2], Johana Bene [4], Pascale Palassin [5], Marina Atzenhoffer [6], Franck Rouby [7], Marion Sassier [8], Marie-Christine Pérault-Pochat [9,10,11], Pascal Roblot [1,2], Marion Allouchery [2,9,†] and Mathieu Puyade [1,11,†] on behalf of the French Network of Regional Pharmacovigilance Centers

[1] Service de Médecine Interne, Centre Hospitalier Universitaire de Poitiers, 86000 Poitiers, France
[2] Faculté de Médecine et de Pharmacie, Université de Poitiers, 86000 Poitiers, France
[3] Institut National de la Santé et de la RechercheMédicale U1313, Université de Poitiers, 86000 Poitiers, France
[4] Centre Régional de Pharmacovigilance du Nord-Pas de Calais, Centre Hospitalier Universitaire de Lille, 59000 Lille, France
[5] Département de Pharmacologie Médicale et Toxicologie, Centre Hospitalier Universitaire de Montpellier, 34295 Montpellier, France
[6] Service Hospitalo, Universitaire de Pharmaco-Toxicologie, Hospices Civils de Lyon, 69424 Lyon, France
[7] CRPV Marseille Provence Corse, Service Hospitalo, Universitaire de Pharmacologie Clinique et Pharmacovigilance, Assistance Publique Hôpitaux de Marseille, 13385 Marseille, France
[8] Département de Pharmacologie, Centre Hospitalier Universitaire de Caen, 14033 Caen, France
[9] Pharmacologie Clinique et Vigilances, Centre Hospitalier Universitaire de Poitiers, 86000 Poitiers, France
[10] Laboratoire de Neurosciences Expérimentales et Cliniques, Institut National de la Santé et de la Recherche Médicale U1084, Université de Poitiers, 86000 Poitiers, France
[11] Centre d'Investigation Clinique-1402, Centre Hospitalier Universitaire de Poitiers, 86000 Poitiers, France
* Correspondence: mickael.martin@univ-poitiers.fr; Tel.: +33-5494-44004.
† These authors contributed equally to the work.

1. Methods S1. Diagnosis and response criteria for ICI-related cytopenias.

1.1 Auto-immune hemolytic anemia (AIHA)

AIHA was defined as hemoglobin (Hb) level <10.0 g/dL, with reticulocyte count >120 G/L, signs of hemolysis (lactate dehydrogenase and/or bilirubin elevation and /or decreased haptoglobin), positive direct antiglobulin test (DAT) and absence of schizocytes. In case of incomplete work-up, improvement after glucocorticoids (GC) and/or no transfusion recovery was in favor of AIHA.

Complete response (CR) was defined as Hb level >11 g/dL (women) or >12 g/dL (men) without features of ongoing hemolysis (including normal haptoglobin level), without any ongoing treatment for AIHA on two different occasions 4-weeks apart, in the absence of any recent transfusion [11]. CR could be considered even with a positive direct antiglobulin test.

Partial response (PR) was defined as Hb level >10 g/dL with at least a 2 g/dL increase from baseline (i.e., at AIHA diagnosis) without any treatment other than GC given at a daily dose >10 mg of prednisone-equivalent or recent transfusion [11].

1.2 Immune thrombocytopenic purpura (ITP)

ITP was defined according to Rodegherio et al [12]. Bone marrow evaluation was needed to exclude hypocellularity or bone marrow infiltration by cancer cells. In case of incomplete work-up, thrombocytopenia <100 G/L without response to platelet transfusion but response to GC and/or to intravenous immunoglobulins was in favor of ITP.

CR was defined as platelet count >100 G/L, without any bleeding event [12]. PR was defined as platelet count >30 G//L with at least 2-fold increase of the baseline count and without any bleeding event [12].

1.3. Auto-immune neutropenia (AIN)

AIN was defined as neutrophil count <1 G/L with normal bone marrow evaluation [13]. In case of incomplete work-up (mostly bone marrow aspiration (BMA) or biopsy (BMB)), chronic (>1 month) and isolated neutropenia without any other cytopenia and etiology was in favor of AIN. Granulocyte colony-stimulating-factor response was NOT considered as part of diagnosis criteria for AIN.

CR was defined as absolute neutrophil count (ANC) >1 G/L [13].

PR was defined as ANC >0.5 G/L with at least 2-fold increase of the baseline count [13].

1.4. Pure red cell aplasia (PRCA)

PRCA was defined as normocytic non-regenerative anemia associated with severe reticulocytopenia and medullar erythroid precursors <5% [14]. Negative Parvovirus B19 IgM serology was another argument in favor of immune-related PRCA. In case of absence of BMA or BMB, chronic (>1 month) reticulocytopenia without any iron or vitamin deficiency and negative Parvovirus B19 serology was in favor of PRCA.

CR was defined as Hb level >10 g/dL without any transfusion.

PR was defined as Hb level between 8 and 10 g/dL with transfusion independence.

1.5. Aplastic anemia (AA)

AA was defined as peripheral cytopenia related to central aplasia (bone marrow granularity <30% according to age). Severity was related to hypogranularity on the BMB <25% associated with at least 2 criteria out of 3: ANC <0.5 G/L, platelet count <20 G/L, reticulocyte count <20 G/L [15].

CR was defined as Hb level >10 g/dL and ANC >1 G/L and platelet count >100 G/L.

PR was defined as transfusion independence with a blood lineage that does not meet diagnosis criteria of severe AA [16].

Table S1. Detailed characteristics of ICI-related cytopenias (*n*=68).

Patient	Age	Sex	Cancer	ICI	Type of immune-related cytopenia	BM analysis	Arguments in favor of diagnosis of immune-related cytopenia	Time to onset (days)
1	65	M	Mel	Ipilimumab	AIHA	NA	Hemolytic anemia, no schizocytes, no transfusion recovery	4
2	77	M	RCC	Nivolumab	AIHA	NA	Hemolytic anemia, DAT (+)	237
3	86	F	RCC	Nivolumab	ITP	Normal BMA	Normal BMA and GC response	27
4	70	M	PC	Nivolumab	AA	BMB: aplasia	Peripheral aplasia, BMB conclusion	131
5	75	M	PC	Nivolumab	ITP	ND	GC response within 72h	60
6	69	F	Mel	Ipilimumab	ITP	ND	No transfusion recovery, MAIPA (+)	67
7	82	M	Mel	Pembrolizumab	PRCA	BMA: erythroblastopenia	BMA, negative parvovirus B19 serology	41
8	66	F	Mel	Pembrolizumab	ITP	ND	GC response within 72h	532
9	42	F	Mel	Nivolumab	PRCA	ND	Reticulocytopenia, absence of hemolysis, transfusion recovery and negative screening for viral causes	65
10	77	M	PC	Nivolumab	AIHA	None	Hemolytic anemia, DAT (+)	399
11	58	M	PC	Nivolumab	ITP	ND	No transfusion recovery, complete response with GC and IVIg	30
12	50	M	PC	Nivolumab	ITP	Normal BMA	Thrombocytopenia and normal BMA	5
13	63	F	Mel	Nivolumab	ITP	Normal BMA	Thrombocytopenia and normal BMA	420
14	72	M	Mel	Nivolumab/ipilimumab	AIHA	NA	Hemolytic anemia, DAT (+)	84
15	66	M	MC	Avelumab	AIHA<br>ITP	NA<br>Normal BMA	Hemolytic anemia, GC response<br>Thrombocytopenia and normal BMA	10<br>UNK
16	59	M	PC	Nivolumab	ITP	ND	No response to platelet transfusion	217
17	63	M	PC	Nivolumab	AIHA	NA	Hemolytic anemia, no schizocytes	35
18	54	M	RCC	Nivolumab	ITP<br>AIN	Normal BMA	Thrombocytopenia and normal BMA<br>Neutropenia and normal BMA	85<br>134
19	61	M	RCC	Nivolumab	ITP	Normal BMA	Thrombocytopenia and normal BMA	268
20	59	F	PC	Nivolumab	ITP	ND	Thrombocytopenia, GC response, persistence of neutropenia following nivolumab discontinuation	77
21	67	M	PC	Atezolizumab	ITP	ND	Thrombocytopenia, GC response within 48h	21
22	58	M	Mel	Pembrolizumab	AIN	Normal BMA	Neutropenia with normal BMA	14

23	74	F	Mel	Nivolumab/ipili-mumab	AIN	Normal BMA	Neutropenia with normal BMA	36
24	68	M	Mel	Nivolumab/ipili-mumab	AA	BMB: aplasia	Peripheral aplasia, BMB conclusion	153
25	41	F	Mel	Nivolumab/ipili-mumab	PRCA	BMA: erythro-blastopenia	BMA, negative parvovirus B19 serology	36
26	61	M	PC	Pembrolizumab	ITP	Normal BMA	Thrombocytopenia and normal BMA	26
27	77	M	UTC	Atezolizumab	AIHA	NA	Hemolytic anemia, DAT (+)	18
28	56	F	Mel	Pembrolizumab	PRCA	BMA: erythro-blastopenia	BMA	140
29	59	M	UTC	Pembrolizumab	ITP	Normal BMA	Thrombocytopenia and normal BMA	77
30	68	F	Mel	Pembrolizumab	AIN	Normal BMA	Neutropenia and normal BMA	62
31	34	F	NHL	Pembrolizumab	AIHA	NA	Hemolytic anemia, DAT (+)	32
32	59	M	PC	Pembrolizumab	ITP	Normal BMA	Thrombocytopenia and normal BMA	459
33	78	F	PC	Pembrolizumab	AIHA	NA	Hemolytic anemia, DAT (+)	5
34	72	M	PC	Durvalumab	ITP	ND	GC response	13
35	59	F	PC	Pembrolizumab	AIN	Normal BMA	Neutropenia and normal BMA	20
					ITP		Thrombocytopenia and normal BMA	20
36	68	M	Mel	Nivolumab/ipili-mumab	AIHA	NA	Hemolytic anemia, DAT (+)	409
					ITP	Normal BMA	Thrombocytopenia and normal BMA	409
37	43	M	PC	Pembrolizumab	ITP	Normal BMA	Thrombocytopenia and normal BMA	18
38	58	M	PC	Pembrolizumab	ITP	ND	GC response	8
39	76	F	HL	Nivolumab	AIHA	NA	Hemolytic anemia, GC response	27
40	55	F	Mel	Nivolumab	AIHA	NA	Hemolytic anemia, DAT (+)	84
					PRCA	BMA: erythro-blastopenia	BMA, negative parvovirus B19 serology	84
41	73	M	Mel	Nivolumab	AIHA	NA	Hemolytic anemia, GC response	60
42	59	F	PC	Pembrolizumab	AIN	Normal BMA	Neutropenia and normal BMA	381
43	83	M	CHC	Atezolizumab	ITP	Normal BMA	Thrombocytopenia and normal BMA	12
44	65	F	PC	Pembrolizumab	ITP	Normal BMA	Thrombocytopenia and normal BMA	27
45	68	M	PC	Pembrolizumab	ITP	Normal BMA	Thrombocytopenia and normal BMA	126
46	34	M	Mel	Pembrolizumab	ITP	Normal BMA	Thrombocytopenia and normal BMA	21
47	60	M	PC	Nivolumab	AIN	Normal BMA	Neutropenia and normal BMA	82
48	78	M	HNSCC	Nivolumab	AIHA	NA	Hemolytic anemia, GC response	53
					ITP	ND	GC response within 48h	53
49	52	F	HNSCC	Nivolumab	AIN	Normal BMA	Neutropenia and normal BMA	279
50	67	F	Mel	Pembrolizumab	ITP	ND	GC response within 48h	428
51	53	M	HNSCC	Nivolumab	ITP	Normal BMA	Thrombocytopenia and normal BMA	41

52	70	M	PC	Nivolumab	PRCA	BMA: erythro-blastopenia	BMA conclusion, negative parvovirus B19 serology	62
53	59	M	PC	Pembrolizumab	ITP	Normal BMA	Thrombocytopenia and normal BMA	46
54	71	F	PC	Pembrolizumab	AIHA	NA	Hemolytic anemia, DAT (+)	353
55	63	M	Mel	Pembrolizumab	AIN	Normal BMA	Neutropenia and normal BMA	61
56	62	M	PC	Pembrolizumab	AIHA	NA	Hemolytic anemia, DAT (+)	43
					ITP	Normal BMA	Thrombocytopenia and normal BMA	43
57	55	F	PC	Pembrolizumab	ITP	Normal BMA	Thrombocytopenia and normal BMA	43
58	51	F	Mel	Pembrolizumab	ITP	ND	GC response within 24h	145
59	65	M	PC	Pembrolizumab	ITP	Normal BMA	Thrombocytopenia and normal BMA	60
60	54	M	PC	Pembrolizumab	AIN	ND	Chronic neutropenia without any other suspected drug	85
61	66	M	PC	Pembrolizumab	ITP	ND	GC and IVIg response within 24h	961
62	53	M	PC	Pembrolizumab	ITP	Normal BMA	Thrombocytopenia and normal BMA	62
63	55	F	PC	Pembrolizumab	ITP	Normal BMA	Thrombocytopenia and normal BMA	31
64	58	F	Mel	Nivolumab/ipili-mumab	AIHA	NA	Hemolytic anemia, DAT (+)	23
65	69	M	PC	Pembrolizumab	ITP	Normal BMA	Thrombocytopenia and normal BMA	192
66	71	M	Met Ep	Nivolumab	AIHA	NA	Hemolytic anemia, DAT (+)	11
67	71	F	Mel	Pembrolizumab	AIHA	NA	Hemolytic anemia, GC response	67
68	59	M	PC	Pembrolizumab	ITP	ND	GC response within 72h	19

AA: Aplastic anemia, AIHA: Autoimmune hemolytic anemia, AIN: Autoimmune neutropenia, BM: Bone marrow, BMA: Bone marrow aspiration, BMB: Bone marrow biopsy, DAT: Direct antiglobulin test, F: Female, GC: Glucocorticoids, HCC: Hepatocellular carcinoma, HNSCC: Head and neck squamous cell carcinoma, ICI: Immune checkpoint inhibitor, IVIg: Intravenous immunoglobulins, ITP: Immune thrombocytopenic purpura, M: Male, NA: Not applicable, NHL: Non-Hodgkin lymphoma, MAIPA: Monoclonal antibody-specific immobilization of platelet, Mel : Melanoma, Met Ep: Metastatic epidermoid cancer of unknown origin, ND: Not done, PRCA: Pure red cell aplasia, PC: Pulmonary cancer, RCC: Renal cell carcinoma, UNK: Unknown, UTC: Urinary tract cancer.
